# Supplementary material for: Single Nucleotide Polymorphisms as Biomarker Predictors of Oral Mucositis Severity in Head and Neck Cancer Patients Submitted to Combined Radiation Therapy and Chemotherapy: A Systematic Review
Source: Cancers (Basel). 2024 Feb 27;16(5):949. doi: 10.3390/cancers16050949 (PMC10931025; doi:10.3390/cancers16050949)
Supplement: Supplementary file 1 [file cancers-16-00949-s001.zip › cancers-2821234-supplementary.pdf]

**Table S1.** Risk of bias assessment

| Studies | Selection 1 | Selection 2 | Selection 3 | Selection 4 | Comparability 1 | Results 1 | Results 2 | Results 3 | Total Points |
|---|---|---|---|---|---|---|---|---|---|
| Werbrouck 2009 [13} | * | 0 | * | 0 | * | * | * | * | 6 |
| Patresi 2011 [14] | * | 0 | * | 0 | 0 | * | * | * | 5 |
| Li 2013 [15} | * | 0 | * | 0 | 0 | * | * | * | 5 |
| Venkatesh 2014 [16] | * | 0 | * | 0 | 0 | * | * | * | 5 |
| Yu 2016 [17] | * | 0 | * | 0 | 0 | * | * | 0 | 4 |
| Chen 2017 [19] | * | 0 | * | 0 | 0 | * | * | * | 5 |
| Guo 2017 [20] | * | 0 | * | 0 | 0 | * | * | * | 5 |
| Guo 2017 lng [18] | * | 0 | * | 0 | 0 | * | * | 0 | 4 |
| Le Z 2017 [21] | 0 | 0 | * | 0 | 0 | * | * | * | 4 |
| Borchiellini 2017 [24] | * | 0 | * | 0 | 0 | * | * | * | 5 |
| Reyes-Gibby 2017 [23] | * | 0 | * | 0 | * | * | * | * | 6 |
| Brzozowska 2018 TNF [27] | * | 0 | * | * | 0 | * | * | * | 6 |
| Brzozowska 2018 [26] | * | 0 | * | 0 | * | * | * | * | 6 |
| Nanda 2018 [25] | * | 0 | * | 0 | 0 | * | * | * | 5 |
| Duran 2019 [28] | * | 0 | * | 0 | 0 | * | * | * | 5 |
| Yang 2019 [29] | * | 0 | * | 0 | 0 | * | * | * | 5 |
| Gupta 2019 [30] | * | 0 | * | * | 0 | * | * | * | 6 |
| Mlak TNFR 2020 [31] | 0 | 0 | * | * | 0 | * | * | * | 5 |
| Mlak TNF Alfa 2020 [32] | 0 | 0 | * | * | 0 | * | * | * | 5 |
| Yang 2020 [33] | * | 0 | * | 0 | * | * | * | * | 6 |
| Raturi 2020 [34] | * | 0 | * | 0 | * | * | * | * | 6 |
| Quinghua 2021 [35] | * | 0 | * | 0 | * | * | * | * | 6 |
